# Efficacy of oral fluralaner for the treatment of canine generalized demodicosis: a molecular-level confirmation

**DOI:** 10.1186/s13071-019-3521-9

**Published:** 2019-05-28

**Authors:** Milos Djuric, Natalija Milcic Matic, Darko Davitkov, Uros Glavinic, Dajana Davitkov, Branislav Vejnovic, Zoran Stanimirovic

**Affiliations:** 1 Department of Equine, Small Animal, Poultry and Wild Animal Diseases, Faculty of Veterinary Medicine, University of Belgrade, Bul. oslobodjenja 18, 11000 Belgrade, Serbia; 2 Department of Biology, Faculty of Veterinary Medicine, University of Belgrade, Bul. oslobodjenja 18, 11000 Belgrade, Serbia; 3 Department of Forensic Veterinary Medicine, Faculty of Veterinary Medicine, University of Belgrade, Bul. oslobodjenja 18, 11000 Belgrade, Serbia; 4 Department of Economics and Statistics, Faculty of Veterinary Medicine, University of Belgrade, Bul. oslobodjenja 18, 11000 Belgrade, Serbia

**Keywords:** *Demodex canis*, Dog, Fluralaner, Real-time PCR, Treatment

## Abstract

**Background:**

Canine generalized demodicosis is a common parasitic disease caused by the proliferation of *Demodex* mites. The introduction of isoxazoline class treatments in veterinary dermatology has resulted in apparently effective treatment of generalized demodicosis. The objective of this study was to evaluate the effectiveness of fluralaner for the treatment of canine generalized demodicosis using real-time PCR for the detection and quantification of *Demodex* DNA.

**Methods:**

Twenty privately owned dogs with clinical symptoms of generalized demodicosis and deep skin scrapings positive for *Demodex canis* mites were enrolled in the study. Following diagnosis (day 0) each dog was treated with fluralaner at the recommended commercial dose for tick and flea treatment (25–56 mg/kg) based on body weight. Clinical and mite count assessments, and hair sampling for molecular analyses were performed on days 0, 28, 56, 84 and 112. *Demodex* DNA was detected and quantified using real-time PCR.

**Results:**

A single oral dose of fluralaner reduced *Demodex* mite counts in skin scrapings by an average of 98.9% in all dogs by day 28. No mites were recovered from skin scrapings from any treated dog by day 56, at which time the dog was considered to be clinically cured, with total hair regrowth. There were significant differences among examined dogs in qPCR cycle threshold (Ct) values on days 0, 28, 56, 84 and 112. *Demodex* DNA levels decreased (increasing Ct values) throughout the study. Mite DNA was present on day 112, possibly from dead mites, at values significantly lower than in samples taken on days 0, 28 and 56. Based on qPCR testing of diluted samples, the *Demodex* mite population was reduced by approximately 1000-fold on day 112.

**Conclusions:**

Oral administration of fluralaner at the recommended dose to dogs with generalized demodicosis is highly effective for reducing *Demodex* mite populations and resolving clinical signs of generalized demodicosis. The presence of mite DNA may indicate that treatment did not kill all *Demodex* mites.

## Background

Canine generalized demodicosis is a common inflammatory parasitic skin disease of dogs associated with the proliferation of *Demodex* mites in hair follicles and sebaceous glands [1, 2]. This disease remains one of the most difficult treatment challenges in veterinary dermatology [3]. Dogs can be affected by three recognized *Demodex* species: *D. canis*, *D. injai* and *D. cornei* [4–6]. There is still controversy whether *D. cornei* is a new species, and further studies are required to ascertain the specific status of this mite [7]. Demodicosis can be classified as local or generalized and adult or juvenile onset depending on the extent of the lesions and the age of the affected dog. Demodicosis can be considered localized if there are no more than four lesions and none larger than 2.5 cm in diameter [8]. A definitive diagnosis is established through finding numerous adult mites and/or immature forms on microscopic examination of deep skin scrapings, hair plucking, or acetate tape impressions obtained from affected areas. Hair plucking and acetate tape impressions are less useful once treatment is administered and only skin scrapings can be used for therapeutic monitoring [1].

Demodicosis therapy is time-consuming and frustrating for both dog owners and veterinarians. The only products labelled for treatment of generalized demodicosis in most countries are based on either amitraz or a combination of imidacloprid and moxidectin. Isoxazoline derivatives are a new class of potent ectoparasiticides and include fluralaner, afoxolaner, lotilaner and sarolaner [9–12]. Following their introduction for treating flea and tick infestations, they have been evaluated off-label for generalized demodicosis treatment [13] and several studies report that they are effective for this use [7, 12–17]. The great advantages of isoxazolines are their easy administration and certainty of reaching all body areas [16].

Real-time PCR based on an amplified fragment of the chitin synthase gene is a specific and sensitive technique to detect *Demodex* DNA and a useful tool for epidemiological studies on this mite [3, 18]. The goal of this study was to monitor the effectiveness of fluralaner treatment against canine generalized demodicosis, using real-time PCR to detect and quantify *Demodex* DNA, and to examine whether the fluralaner is able to reduce the *Demodex* population below the detection limit

## Methods

### Dogs and sampling procedures

Twenty privately owned dogs with clinical symptoms of generalized juvenile demodicosis and multiple positive deep skin scrapings for *Demodex* spp. mites were enrolled in this study between January 2016 and November 2017. All dogs were admitted to the Teaching Hospital at the Faculty of Veterinary Medicine in Belgrade for medical consultation and assistance. Relevant data (age, breed, gender and clinical signs) on the patients were collected and recorded by veterinarians during their clinical examination. Most dogs (*n* = 11) were mixed breed, while pure breeds included two Doberman Pinschers and one each of: Pug, English Bulldog, French Bulldog, Maltese, Kangal, Pitbull and Shar Pei. Their age ranged from 3 months to 3 years, with 9 male and 11 female dogs.

On the day of initial diagnosis (day 0) all dogs were treated with fluralaner (Bravecto chewable tablets, MSD Animal Health, Madison, NJ, USA) at the commercial dose (25–56 mg/kg) based on body weight. Enrolled dogs were not treated with glucocorticoids, other ectoparasiticides or macrocyclic lactones for at least one month prior to day 0. Clinical assessment, mite counts and hair sampling for molecular analyses were performed on days 0, 28, 56, 84 and 112. Hair re-growth assessment was performed by comparing alopecic areas over the 16-week study and was expressed as hair re-growth percentage classified as 0–50%, 50–90% or > 90%. Other skin lesions (erythema, scales, follicular casts and crusts) were also assessed in the same way. Photographs illustrating the extent of lesions were taken on each visit to document the changes observed.

Deep skin scrapings were taken at each examination from five affected skin locations, in the direction of hair growth using a sharp blade until capillary bleeding occurred. The scraping was transferred to a marked microscope slide, mixed with mineral oil and examined microscopically at 10× magnification to count adult and immature mites. Mite numbers in each scraping were recorded separately.

Composite hair samples were obtained for molecular analysis by gently plucking hairs in the direction of growth to include the hair roots. Samples were collected from 20 areas including: lip and periocular (four points), perinasal, temporal, chin, ventral and dorsal neck, dorsum (two points), chest, abdomen (two points), thighs (two points) and the interdigital area (four points, one on each foot). Hair samples were maintained in phosphate-buffered saline and stored at − 20 °C until DNA extraction. This sampling method is reliable for detecting *Demodex* spp. on affected dogs [14].

### DNA extraction and real-time PCR

For DNA extraction, samples were centrifuged in a microcentrifuge at 13,000× *rpm* for 30 min. Once the supernatant was removed, samples were submerged in liquid nitrogen and then disrupted using a homogenizer. Isolation was performed using a commercial kit (Thermo Scientific GeneJET Genomic DNA Purification Kit, cat. no. K0722; Thermo Fisher Scientific, Waltham, Massachusetts, USA) according to the manufacturer’s instructions with minor modifications (samples were incubated for 24 h with Proteinase K Solution and Digestion Solution).

Real-time PCR [14] reactions were performed (Rotor-Gene Q 5plex; Qiagen, Hilden, Germany) in a total volume of 20 μl (FastStart Universal SYBR Green Master; Roche Diagnostics GmbH, Mannheim, Germany) using 0.3 µmol/l of each primer and 4 µl of DNA. The following primers were used: *D. canis* forward, 5′-GAT GAA GCG GCG AGT AAT GTT C-3′, reverse, 5′-GAC TCC ATC TTT TAC GAT GTC TGA TTT-3′. These amplified a 166-bp fragment of the chitin synthase gene. Nuclease-free water was used as a negative control for the PCR. Positive controls were obtained from clinical samples that were previously amplified and sequenced to confirm *Demodex*. The thermal cycling profile was as follows: 50 °C for 2 min and 95 °C for 10 min, followed by 40 cycles at 95 °C for 15 s and 60 °C for 1 min.

### Statistical analysis

Data were tested for normality using the Kolmogorov-Smirnov test. Normality was rejected (Kolmogorov-Smirnov test, *P* < 0.05) and the Friedmanʼs test with a *post-hoc* test (Dunn’s pairwise test with Bonferroni correction) were used. Data are presented as median Ct values with corresponding interquartile range (IQR) (25th and 75th percentile). Significant differences were estimated with *P* < 0.05, *P* < 0.01, *P* < 0.001 and *P* < 0.0001 significance levels. Statistical analysis was performed with GraphPad Prism v.6 (GraphPad, San Diego, CA, USA).

## Results

All enrolled dogs completed the study following treatment with the recommended fluralaner dose. All dogs ate the tablet with no evidence of any post-treatment emesis in any dog. No other adverse reactions were observed in any treated dog.

### Clinical symptom evaluation and mite counts

Significant clinical improvement with a dramatic reduction in mite numbers in skin samples was seen by day 28 (Table 1). A single oral dose of fluralaner resulted in an average *Demodex* mite count reduction of 98.9% in all dogs on skin scrapings at the first follow-up visit. On day 56 and on all further examination visits, no mites were recovered on skin scrapings from any treated dog. All patients have reported on dermatological examination a one year after fluralaner therapy, with no clinical signs of demodicosis and no mites were found on skin scrapings.

**Table 1:** Changes in mite counts and clinical signs in dogs with generalized demodicosis following oral treatment with fluralaner

Study day	No. of mites (geometric mean)	Efficacy (%)	Reduction of clinical symptoms and hair regrowth (%)
0	496	–	–
28	5.6	98.9	50–90
56	0	100	> 90
84	0	100	> 90
112	0	100	> 90

**Fig. 1:**
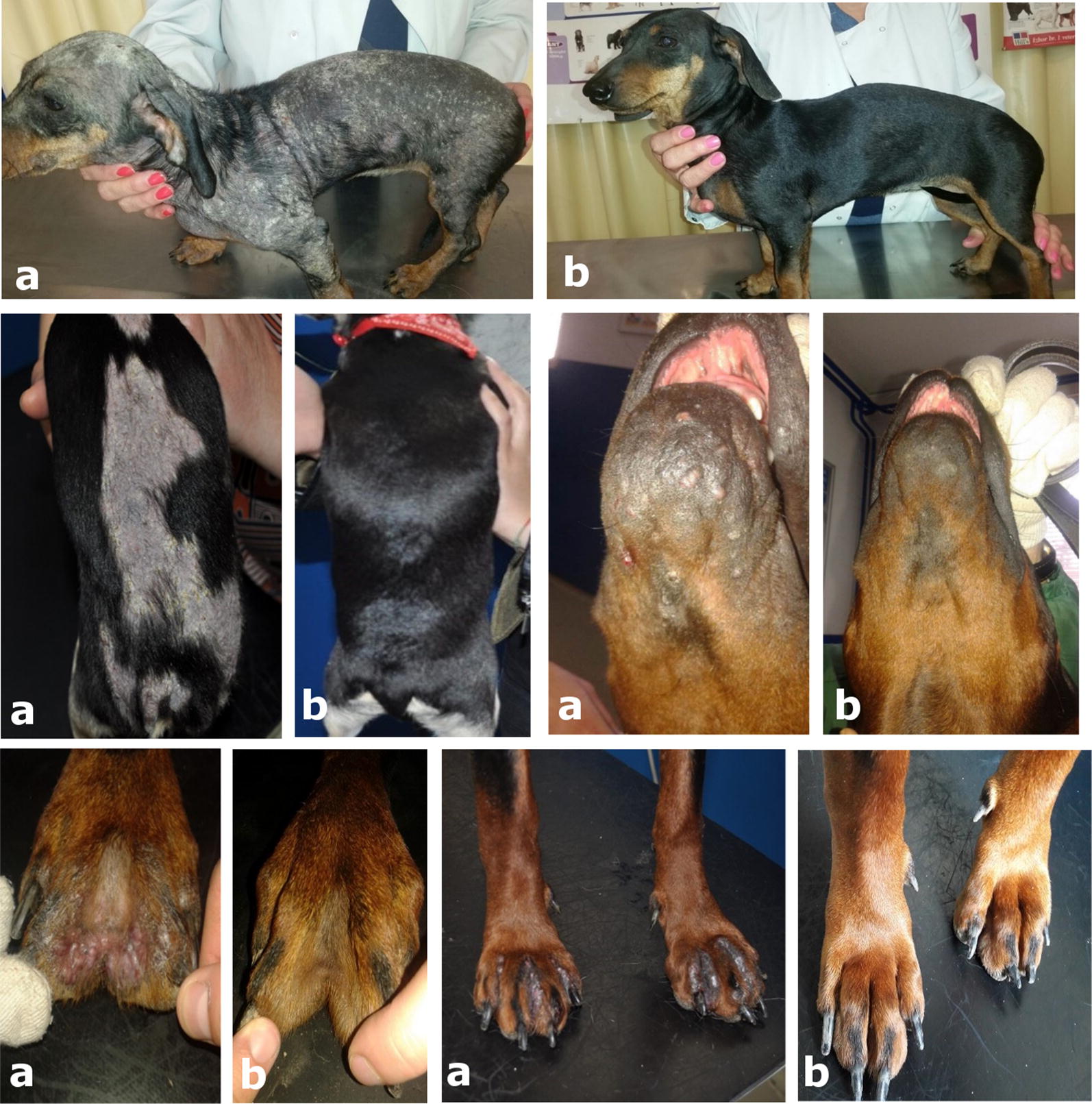
Multiple photos of a dog with generalized demodicosis showing the clinical condition on **a** day 0 and **b** day 56 following fluralaner treatment

Clinical signs of demodicosis also improved remarkably during the first four weeks. Crusts were present on the skin of seven dogs and six dogs still had some erythematous patches of alopecia on day 28. On day 56 all treated dogs showed a complete clinical cure with total hair regrowth (Fig. 1).

### PCR results

**Fig. 2:**
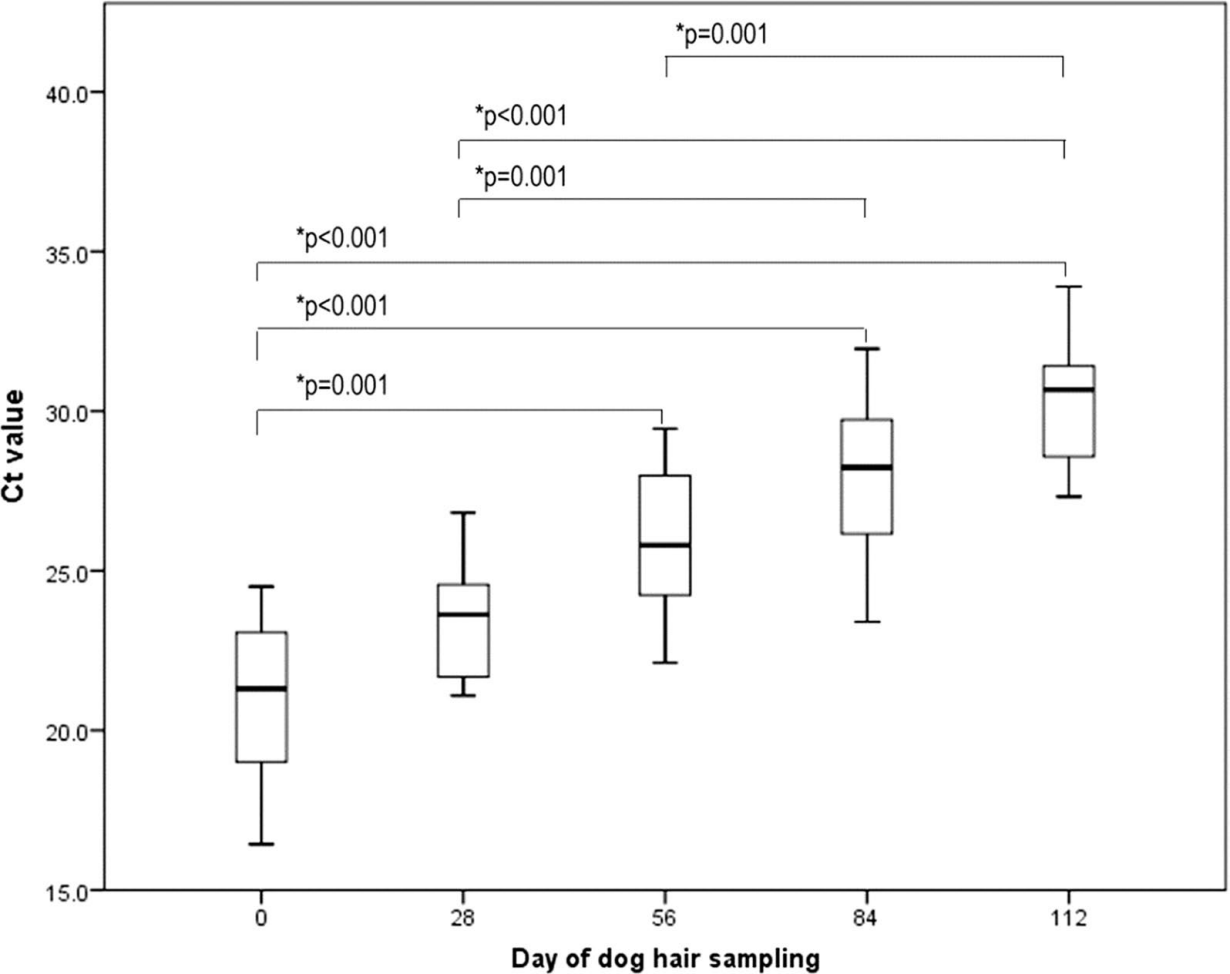
Cycle threshold (Ct) values for qPCR results of *Demodex* mite DNA in samples from dogs with generalized demodicosis treated with fluralaner. Increasing Ct values are consistent with reducing DNA levels

Results of qPCR analyses are presented as cycle threshold (Ct) values for each sample. There were significant differences between Ct values on days 0, 28, 56, 84 and 112 among examined dogs (Friedmanʼs test, *n* = 20, *χ*^2^ = 80.0, *df* = 4, *P* < 0.0001). Median Ct values with corresponding interquartile range (IQR) (25th and 75th percentile) are presented for each time point (Table 2). Dunn’s pairwise *post-hoc* test with Bonferroni correction revealed significant differences in Ct values between days 0 and 56 (*P* = 0.001); 0 and 84 (*P* < 0.001); 0 and 112 (*P* < 0.001); 28 and 84 (*P* = 0.001); 28 and 112 (*P* < 0.001); and 56 and 112 (*P* = 0.001). Ct values were not significantly different between adjacent time points (days 0 and 28, 28 and 56, 56 and 84, and 84 and 112) (Fig. 2).

**Table 2:** qPCR median cycle threshold (Ct) values with interquartile ranges (IQR) for *Demodex* mite DNA in samples from dogs with generalized demodicosis treated with fluralaner on day 0, 28, 56, 84 and 112

Sample	Median Ct (IQR)
Sampled on day 0	21.3 (18.9–23.1)
Sampled on day 28	23.6 (21.7–24.6)
Sampled on day 56	25.8 (24.2–28.1)
Sampled on day 84	28.2 (26.0–29.8)
Sampled on day 112	30.7 (28.4–31.4)
Undiluted control	23.4 (23.4–23.5)
10× diluted control	26.5 (26.4–26.6)
100× diluted control	29.7 (29.5–30.1)

## Discussion

Fluralaner treatment of dogs with generalized demodicosis, a serious and potentially life-threatening disease, resulted in a significant decrease in quantitated *Demodex* DNA. Previously approved treatments require repeated applications over long periods of time, with the potential for adverse side effects [19, 20]. Parasitological healing is considered to be achieved if two consecutive skin scrapings taken at monthly intervals are negative for all *Demodex* life forms, whether dead or alive [21]. In this study, a single oral dose of fluralaner resulted in negative skin scrapings in all treated dogs by day 56, and all dogs remained negative on all further skin scrapings. On day 112, skin scrapings from all the dogs in this study remained negative for *Demodex* mites (Table 1), despite being four-weeks beyond the protection period against flea and tick infestation [22].

All dogs enrolled in this study had clinical signs of generalized demodicosis at the time of treatment (day 0) including: erythematous patches, alopecia, scales, crusts and follicular casts. The decrease in mite counts observed in the skin scrapings was paralleled by a dramatic reduction in the extent and severity of the skin lesions. Eight weeks after fluralaner treatment all dogs were without clinical signs of demodicosis and total hair regrowth was observed. Our results are consistent with research undertaken by Fourie et al. [7].

The qPCR revealed a decrease in the amounts of *Demodex* spp. DNA (increasing Ct values) throughout the post-treatment period. *Demodex* DNA was still detectable on day 112 but was significantly lower in comparison with samples from days 0, 28 and 56 (Fig. 1). By using molecular methods to detect *Demodex* DNA, it is theoretically possible to amplify DNA from dead mites. However, the epidermis of the dog undergoes continuous renewal with full turnover occurring approximately every 21 days [13, 23], so the detection of dead mite DNA is unlikely. Throughout the whole experiment the quantity of DNA detected by qPCR decreased. This reduction proves that the majority of the DNA load came from live *Demodex*. If the DNA of all dead *Demodex* parasites had remained in the skin, the decrease in DNA and a consequent increase in Ct values throughout the experiment would not be so significant. Ct values were continually rising over time (Table 2) and by day 112, the median Ct value increased by 9.4 cycles in comparison to day 0. Standard serial dilutions of a control sample were included in each run and their Ct values increased by around 3 cycles in each of the following dilutions. Assuming 100% amplification efficiency, it can be concluded that the *Demodex* population was reduced between 100 and 1000 times. Therefore, Fluralaner at the labelled dose may not have completely eliminated the *Demodex* mites although their population was significantly reduced. This is consistent with previous work showing that treatment with afoxolaner and fluralaner does not impact *Demodex* populations in normal dogs [13]. It would be of interest to monitor dogs for a longer period of time, to determine Ct values every four weeks and to compare this results with the Ct values of asymptomatic dogs that never had canine demodicosis.

## Conclusions

Oral administration of fluralaner to dogs with generalized demodicosis at the recommended dose for treating fleas and ticks is highly effective for reducing *Demodex* mite counts and eliminating the clinical signs of disease. A single treatment with fluralaner may not completely eliminate all mites based on qPCR measurement of mite DNA.

## Data Availability

All data generated or analyzed during this study are included in this article.

## Abbreviations

PCR: polymerase chain reaction
DNA: deoxyribonucleic acid
Ct: cycle threshold
IQR: interquartile range
SPC: summary of product characteristics

## References

[1] Miller WH, Griffin CE, Campbell KL, Muller GH (2013). Muller and Kirkʼs small animal dermatology.

[2] Moskvina TV (2017). Two morphologically distinct forms of *Demodex* mites found in dogs with canine demodicosis from Vladivostok, Russia. Acta Vet Beograd.

[3] Ravera I, Altet L, Francino O, Sánchez A, Roldán W, Villanueva S, Bardagí M, Ferrer L (2013). Small *Demodex* populations colonize most parts of the skin of healthy dogs. Adv Vet Dermatol.

[4] Shipstone M (2000). Generalised demodicosis in dogs, clinical perspective. Aust Vet J.

[5] Desch CE, Hillier A (2003). *Demodex injai*: a new species of hair follicle mite (Acari: Demodecidae) from the domestic dog (Canidae). J Med Entomol.

[6] de Rojas M, Riazzo C, Callejón R, Guevara D, Cutillas C (2012). Molecular study on three morphotypes of *Demodex* mites (Acarina: Demodicidae) from dogs. Parasitol Res.

[7] Fourie JJ, Liebenberg JE, Horak IG, Taenzler J, Heckeroth AR, Frénais R (2015). Efficacy of orally administered fluralaner (Bravecto™) or topically applied imidacloprid/moxidectin (Advocate®) against generalized demodicosis in dogs. Parasit Vectors.

[8] Mueller RS, Bensignor E, Ferrer L, Holm B, Lemarie S, Paradis M, Shipstone MA (2012). Treatment of demodicosis in dogs: 2011 clinical practice guidelines. Vet Dermatol.

[9] Ozoe Y, Asahi M, Ozoe F, Nakahira K, Mita T (2010). The antiparasitic isoxazoline A1443 is a potent blocker of insect ligand-gated chloride channels. Biochem Biophys Res Commun.

[10] García-Reynaga P, Zhao C, Sarpong R, Casida JE (2013). New GABA/glutamate receptor target for [3H] isoxazoline insecticide. Chem Res Toxicol.

[11] Gassel M, Wolf C, Noack S, Williams H, Ilg T (2014). The novel isoxazoline ectoparasiticide fluralaner: selective inhibition of arthropod γ-aminobutyric acid-and l-glutamate-gated chloride channels and insecticidal/acaricidal activity. Insect Biochem Mol Biol.

[12] Beugnet F, Halos L, Larsen D, de Vos C (2016). Efficacy of oral afoxolaner for the treatment of canine generalised demodicosis. Parasite.

[13] Zewe CM, Altet L, Lam AT, Ferrer L (2017). Afoxolaner and fluralaner treatment do not impact on cutaneous *Demodex* populations of healthy dogs. Vet Dermatol.

[14] Six RH, Becskei C, Mazaleski MM, Fourie JJ, Mahabir SP, Myers MR, Slootmans N (2016). Efficacy of sarolaner, a novel oral isoxazoline, against two common mite infestations in dogs: *Demodex* spp. and *Otodectes cynotis*. Vet Parasitol.

[15] Snyder DE, Wiseman S, Liebenberg JE (2017). Efficacy of lotilaner (Credelio™), a novel oral isoxazoline against naturally occurring mange mite infestations in dogs caused by *Demodex* spp. Parasit Vectors.

[16] Becskei C, Cuppens O, Mahabir SP (2018). Efficacy and safety of sarolaner against generalized demodicosis in dogs in European countries: a non-inferiority study. Vet Dermatol.

[17] Duangkaew L, Larsuprom L, Anukkul P, Lekcharoensuk C, Chen C (2018). A field trial in Thailand of the efficacy of oral fluralaner for the treatment of dogs with generalized demodicosis. Vet Dermatol.

[18] Ravera I, Altet L, Francino O, Bardagí M, Sánchez A, Ferrer L (2011). Development of a real-time PCR to detect *Demodex canis* DNA in different tissue samples. Parasitol Res.

[19] Johnstone IP (2002). Doramectin as a treatment for canine and feline demodicosis. Aust Vet Pract.

[20] Bissonnette S, Paradis M, Daneau I, Silversides DW (2009). The ABCB1-1Δ mutation is not responsible for subchronic neurotoxicity seen in dogs of non-collie breeds following macrocyclic lactone treatment for generalized demodicosis. Vet Dermatol.

[21] Paterson TE, Halliwell RE, Fields PJ, Louw ML, Ball G, Louw J, Pinckney R (2014). Canine generalized demodicosis treated with varying doses of a 2.5% moxidectin+ 10% imidacloprid spot-on and oral ivermectin: parasiticidal effects and long-term treatment outcomes. Vet Parasitol.

[22] European Commission. Community register of veterinary medicinal products, Product information, Annex 1 Summary of product characteristics Bravecto. 2014. http://ec.europa.eu/health/documents/community-register/html/v158.htm.

[23] Kligman AM, Christensen MS (2011). *Demodex folliculorum*: requirements for understanding its role in human skin disease. J Invest Dermatol.

